# Extracorporeal Cardiopulmonary Resuscitation in Dementia: Neurologically Favorable Survival After 110 Minutes of Resuscitation

**DOI:** 10.7759/cureus.95783

**Published:** 2025-10-30

**Authors:** Nihat Firat Sipahi, Mumi Taleb, Hamzah Adwan, Karsten Leitner, Simon Little

**Affiliations:** 1 Department of Emergency and Intensive Care Medicine, Agaplesion Markus Hospital, Academic Teaching Hospital of the Goethe University, Frankfurt, DEU; 2 Air Rescue, Johanniter-Unfall-Hilfe, Giessen, DEU; 3 Institute of Radiology, Agaplesion Markus Hospital, Academic Teaching Hospital of the Goethe University, Frankfurt, DEU; 4 Department of Trauma and Orthopedic Surgery, BG Trauma Center Frankfurt, Frankfurt, DEU

**Keywords:** air rescue, dementia, ecmo, ecpr, emergency medical services (ems), ohca, prolonged cpr, pulmonary embolism

## Abstract

For selected patients with refractory out-of-hospital cardiac arrest (OHCA), extracorporeal cardiopulmonary resuscitation (eCPR) has emerged as a life-saving option, requiring comprehensive organization and collaboration between emergency medical services (EMS) and eCPR-receiving centers. Although various eCPR criteria have been proposed for refractory OHCA, the decision to initiate eCPR remains highly challenging. We present the case of a female patient with mild cognitive impairment who survived 110 minutes of resuscitation for pulmonary embolism, with a neurologically favorable outcome, achieved through coordinated teamwork between pre-hospital and in-hospital care.

## Introduction

Extracorporeal cardiopulmonary resuscitation (eCPR) has evolved into a recognized therapeutic option for refractory out-of-hospital cardiac arrest (OHCA) and is formally recommended for use in highly selected patients, according to current international resuscitation guidelines [1,2]. In addition to these guideline-based recommendations for highly selected patients, a recent review emphasizes that patient selection for eCPR should be a multidisciplinary team decision, based on a careful weighing of arguments for and against its use, a process that reflects real-world clinical practice [3].

eCPR poses a unique challenge for any healthcare system, as it demands substantial resources and high organizational readiness. As a result, there is considerable variation across eCPR centers in how inclusion criteria are applied. Some programs follow strict protocols to improve outcomes and manage resources efficiently, while others adopt more liberal criteria to offer more patients a chance at survival. This leads to heterogeneous patient populations that are difficult to compare. Recent studies support this observation [4,5]. While stricter criteria may improve survival rates per treated patient, they may also prevent access to potentially life-saving treatment. Withholding such an intervention solely to optimize resource utilization raises important ethical concerns.

Here, we report the case of a 71-year-old female patient with mild cognitive impairment who underwent 110 minutes of cardiopulmonary resuscitation for pulmonary embolism and ultimately achieved a neurologically favorable outcome made possible through close coordination between pre-hospital and in-hospital care teams.

## Case presentation

Emergency medical services (EMS) were dispatched to a nursing home for a 71-year-old woman presenting with severe dyspnea, signs of distress, and cape cyanosis. Initial monitoring revealed an oxygen saturation (SpO₂) of 75% on room air, sinus tachycardia, and a barely palpable pulse. The patient was agitated but responsive and able to answer questions regarding her medical history. She had a history of arterial hypertension, obesity, chronic kidney disease, metabolic syndrome, depression, and mild cognitive impairment, consistent with early-stage dementia. When asked whether intensive care measures were desired, she clearly expressed her wish for full therapeutic intervention.

**Figure 1:**
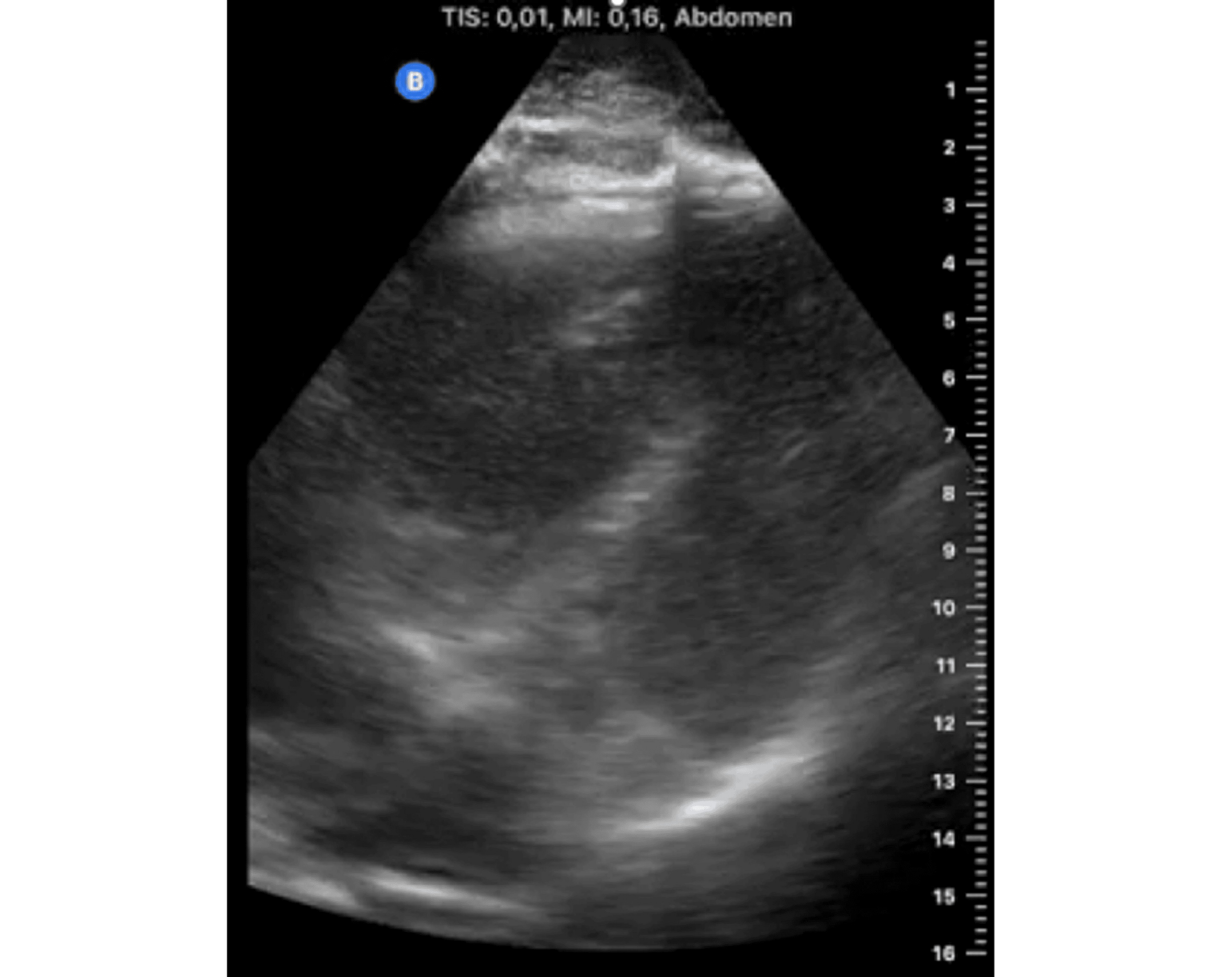
Focused cardiac ultrasonography on scene. Focused cardiac ultrasound performed by the emergency medical services physician revealed signs of severe right ventricular strain, consistent with acute pulmonary embolism. The right ventricle appeared dilated and hypokinetic, while the left ventricle was small and hyperdynamic.

Upon arrival of the EMS physician, the patient showed progressive low cardiac output due to severe right ventricular impairment, as revealed by focused cardiac ultrasonography (Figure 1).

Therefore, immediate cardiopulmonary resuscitation (CPR) was initiated, and 20 mg of alteplase was administered. The patient was endotracheally intubated for sufficient oxygenation. Capnography was used to monitor end-tidal CO₂ as an indicator of CPR quality. Mechanical chest compressions were initiated by the EMS team and continued throughout resuscitation. Return of spontaneous circulation (ROSC) occurred twice but was not sustainable, despite high-dose catecholamine therapy.

In the absence of absolute contraindications and in light of the patient’s clearly stated preference for maximal therapy, she was considered for eCPR and referred to the nearest tertiary care center with extracorporeal membrane oxygenation (ECMO) capability (~55 km distance). Although ECMO availability was marked as “available” in the regional web-based coordination system, the center declined the patient’s candidacy for eCPR and recommended transfer to one of the next-closest centers, located 30-41 km away, all of which were marked as “unavailable” at the time.

Due to the EMS physician’s strong conviction regarding a valid indication for eCPR, based on the patient’s age (71 years), witnessed arrest in the presence of EMS, intermittent ROSC, absence of known life-limiting comorbidities, initial rhythm of pulseless electrical activity, and suspected pulmonary embolism as a potentially reversible cause, our ECMO referral hotline was contacted for consultation. After a rapid assessment of the inclusion criteria, the patient was accepted for eCPR.

**Figure 2:**
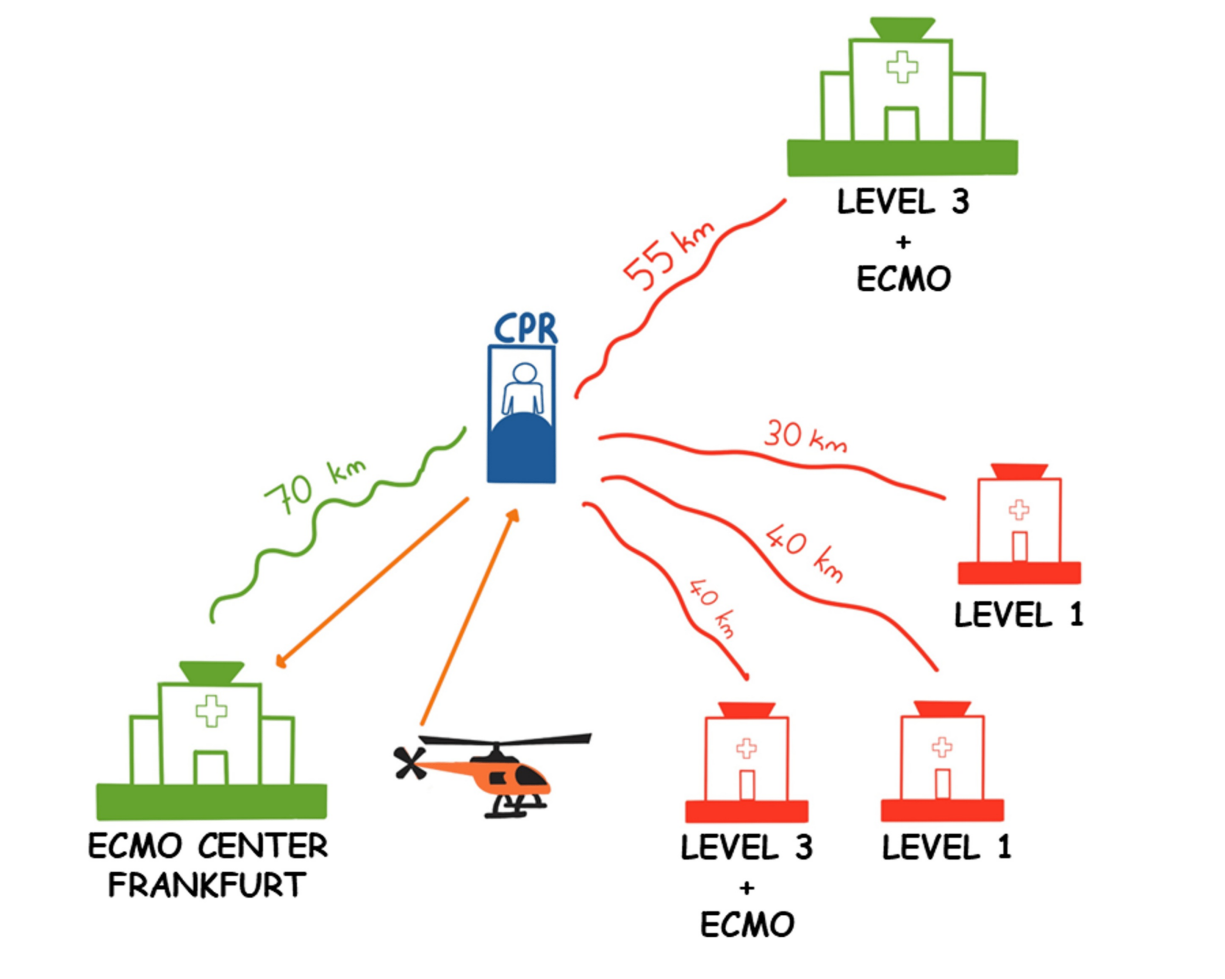
Logistical challenges in regional eCPR access and referral pathway. Illustration of regional eCPR referral process highlighting the logistical burden and access limitations to timely eCPR. Despite ECMO availability (green), the nearest tertiary center declined the patient. Several other potential hospitals were also unavailable (red). Final transport was performed via helicopter to the accepting ECMO center 70 km away. eCPR = extracorporeal cardiopulmonary resuscitation; ECMO = extracorporeal membrane oxygenation

Given the time elapsed since cardiac arrest and the 70 km ground distance to our ECMO center, helicopter transport was arranged to minimize delay (Figure 2).

Twenty minutes after arrival at our ECMO unit, and following a cumulative 110 minutes of CPR, ECMO flow was successfully established via percutaneous cannulation under ultrasound guidance, although cannulation was technically challenging due to the patient’s body habitus (body mass index >38 kg/m²). The patient was subsequently stabilized. Initial laboratory and arterial blood gas values, obtained immediately before ECMO initiation, are summarized in Table 1.

**Table 1:** Initial laboratory and arterial blood gas values. AST = aspartate aminotransferase; ALT: alanine aminotransferase; NT-proBNP = N-terminal of the prohormone brain natriuretic peptide; hsTnT = troponin T high-sensitive; CRP = C-reactive protein; PCT = procalcitonin; BUN = blood urea nitrogen; GFR = glomerular filtration rate; INR: international normalized ratio

Parameter	Patient value	Reference range
pH	7.13	7.35–7.45
pO_2_ (mmHg)	88	75–100
pCO_2_ (mmHg)	58	35–46
Lactate (mg/dL)	53	0–20
Base excess (mmol/L)	-8.8	–2–+2
Bicarbonate (mmol/L)	15.8	22–26
Leukocyte (/nL)	14.3	4.0–10.1
Hemoglobin (g/dL)	9.9	12.0–16.0
Thrombocyte (/nL)	155	140–360
AST (U/L)	405	<35
ALT (U/L)	225	<35
Total bilirubin (mg/dL)	0.3	<1.2
NT-proBNP (pg/mL)	1,559	<285
hsTnT (pg/mL)	376	<14
CRP (mg/L)	7.9	<5
PCT (ng/mL)	0.05	<0.5
Creatinine (mg/dL)	1.48	0.50–0.90
BUN (mg/dL)	71	16.6–48.5
GFR (mL/minute)	35	>60
Quick (%)	27	>70
INR	2.8	0.85–1.15
D-dimer (mg/L)	>20	<0.80

Parameters such as pH and lactate played a supportive role in the pre-implantation clinical assessment and contributed to the decision to proceed with eCPR. Post-implantation laboratory results showed preserved hepatic and renal function, supporting the absence of multiorgan dysfunction and retrospectively validating the appropriateness of this decision.

**Figure 3:**
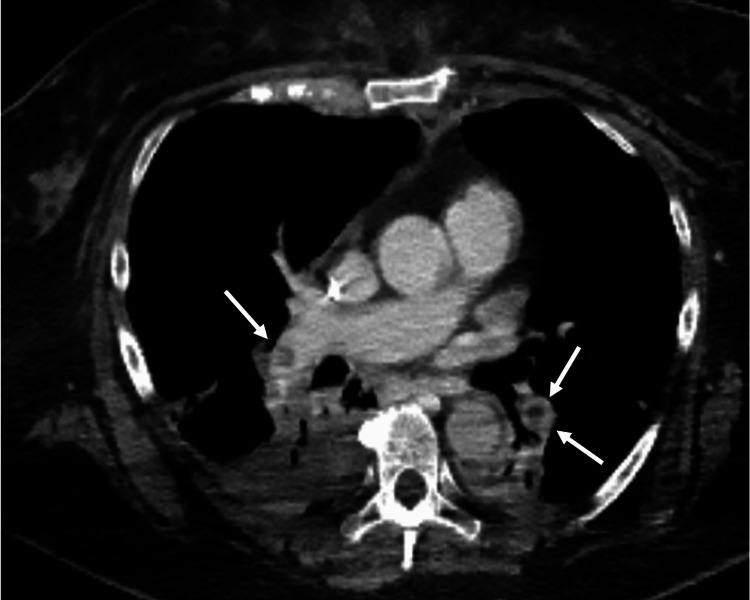
Chest CT scan following ECMO initiation. Axial contrast-enhanced CT image of the chest confirmed bilateral pulmonary embolisms in the right pulmonary artery and in the lobar artery of the left lower lobe (white arrows). ECMO = extracorporeal membrane oxygenation

CT confirmed the suspected pulmonary embolism, with thrombi located in the right pulmonary artery and in the lobar artery of the left lower lobe (Figure 3).

Additionally, bilateral pulmonary infiltrates were diagnosed. Following microbiological sampling, empirical antibiotic therapy with piperacillin/tazobactam was initiated. According to the cardiology consultation, there was no indication for interventional thrombectomy due to the absence of right ventricular (RV) strain under ECMO support, the location of the embolus, and prior administration of thrombolysis. Duplex ultrasonography revealed a non-compressible and thrombosed right femoral vein in its upper third, with the thrombus extending to the mid-segment of the femoral vein. On the left, the popliteal vein was also non-compressible. Later on the same day, transesophageal echocardiography showed no signs of RV overload. The right ventricle appeared no more dilated but showed concentric hypertrophy. With an ECMO flow of 2.7 L/minute at 3,500 rpm, hemodynamic stability was achieved. After a stepwise reduction of catecholamines, a continuous nitroglycerin infusion was initiated to control blood pressure and reduce pulmonary vascular resistance.

A detailed anamnesis obtained through her son revealed a history of prior pulmonary embolism and stroke. The patient had been mobile in her daily life at the nursing home. Although never formally diagnosed, she had been suffering from mild cognitive impairment. Based on this additional information, and after shared decision-making with her son, a ceiling of care was established according to the patient’s presumed wishes: no further escalation (no re-intubation, no re-ECMO, and do-not-resuscitate) in case of future deterioration.

As standard practice, daily wake-up trials were conducted to assess neurological status following ultra-prolonged resuscitation. On ECMO day two, the patient unexpectedly opened her eyes, made eye contact, and showed purposeful movements in all four limbs.

After successful weaning, ECMO was surgically explanted; the procedure proved technically challenging due to arterial injury. A dissection and partial circumferential tear of the common femoral artery required resection and vascular reconstruction using a tailored pericardial tube graft, followed by successful end-to-end anastomosis with restored distal perfusion.

The postoperative course was uneventful. The patient was successfully weaned from mechanical ventilation and extubated on postoperative day zero. On intensive care unit (ICU) day 11, she was transferred to our geriatric rehabilitation department. During the further course, a superficial wound healing disorder of the ECMO access site developed, with no signs of infection or microbial colonization. Plastic surgery consultation recommended secondary intention healing. After a 26-day hospital stay and improved functional status, she was discharged back to the nursing home. Six months after eCPR, the patient remains clinically stable and well in her familiar nursing home environment. She is mobile with a walker and demonstrates a favorable neurological outcome with a Cerebral Performance Category score of 1.

## Discussion

This report presents a septuagenarian who survived an ultra-prolonged resuscitation effort, thanks to the individual decision-making of an EMS physician and the well-coordinated collaboration between EMS, air rescue, and the receiving eCPR center. The case highlights how patient-centered actions and logistical readiness can impact outcomes, even under extreme circumstances.

Balancing clinical benefits with ethical responsibility is particularly complex when it comes to selecting patients for eCPR. In a retrospective multi-regional study, Diehl et al. examined the impact of different selection strategies on survival in eCPR [4]. They found that applying restrictive inclusion criteria was associated with a higher rate of neurologically favorable survival; however, these same criteria would have excluded up to 63% of actual survivors with OHCA [4]. Similarly, Moerk et al. demonstrated that adding stricter thresholds, such as low-flow time under 100 minutes, pH >6.8, lactate <15 mmol/L, or the presence of signs of life, could increase 30-day survival from 30% to 48%, but would have excluded 58% of the real survivors from eligibility [5]. These findings underscore a critical dilemma: while stricter criteria may enhance statistics, they may simultaneously reduce the absolute number of lives saved. This raises serious concerns regarding patient equity and the potential ethical implications of limiting access to a life-saving treatment for the sake of statistical efficiency.

Emergency response to a nursing home may be challenging, especially for life-threatening emergencies. Most residents are chronically ill, frail, or cognitively impaired. On one hand, the presence of caregiving staff often allows responders to quickly gather relevant medical information or identify advanced care plans, making evaluation easier than in other settings. On the other hand, these patients may be unintentionally stigmatized due to their appearance or condition. In critical situations, it can be difficult to imagine that such patients still participate in daily life. In our case, the patient presented with severe dyspnea, signs of significant distress, and cape cyanosis. She had multiple comorbidities and mild cognitive impairment, but she clearly and actively requested medical help. In the absence of contraindications and with a potentially reversible cause of OHCA, she was assessed and accepted for eCPR.

A core principle of ECMO therapy is the concept of a “bridge to decision,” particularly in patients where background information is initially limited [6]. In this case, after establishing ECMO flow and stabilizing the patient, we were able to gather essential insights from her son. He reported that she had previously suffered a pulmonary embolism and had been prescribed direct oral anticoagulants, which had been discontinued, apparently based on external advice. Through this conversation, we also learned more about her functional status and quality of life before the event. At the time of this exchange, the patient had just undergone 110 minutes of resuscitation. Although she had stabilized under ECMO support, her neurological prognosis remained uncertain. In a shared decision-making process with her son, a ceiling of care was defined in accordance with her presumed preferences: no further escalation in case of future clinical deterioration.

The recently published European Society of Intensive Care Medicine consensus recommendations on the management of very old patients in intensive care strongly support this individualized approach [7]. Notably, they do not advocate for rigid exclusion criteria, even in patients aged over 80. Instead, they emphasize that decisions regarding life-sustaining treatment should be made on an individual basis, considering the likely prognosis, the time-dependent burden of intensive interventions, and, crucially, the values and preferences of the patient. During the consensus process, members of the steering group even raised concerns about including the item “current residence” (e.g., living in a nursing home) in assessment checklists, warning that this information could potentially be misused in some healthcare systems to unjustly deny ICU admission. These positions emphasize that, even for very old and frail patients, access to potentially life-saving treatment such as ICU care or eCPR should not be automatically withheld, but rather decided through a balanced, ethically grounded, and individualized approach.

In this case, the initial refusal by the nearest tertiary ECMO center, despite a clearly communicated and valid indication from the EMS physician, illustrates a common problem encountered in systems with restrictive eCPR protocols. This misjudgment resulted in a delay of more than 30 minutes before ECMO flow could be established. Although the patient ultimately had a favorable outcome, such delays are well known to be associated with significantly increased morbidity and mortality in cardiac arrest patients undergoing eCPR [3]. Therefore, this should not be regarded as an acceptable byproduct of restrictive inclusion criteria but rather as a critical system-level failure that may deny patients a realistic chance of survival. This experience highlights the urgent need for improved coordination, awareness, and shared understanding across all levels of care, from EMS to eCPR-providing centers. Only through seamless integration of these elements can we move toward a truly effective and equitable eCPR system. In the future, such systems may be the decisive factor in determining survival after OHCA.

## Conclusions

This report illustrates a successful eCPR case with a neurologically favorable outcome following 110 minutes of resuscitation, made possible through coordinated teamwork between pre-hospital and in-hospital providers. Beyond the clinical achievement, this case highlights the critical importance of inclusion criteria in eCPR programs, which, if applied too rigidly, may risk excluding patients who could otherwise benefit from this life-saving therapy.
